# Diagnostic Utility of Serum Activating Transcription Factor 4 and Toll-like Receptor 4 as Early Biomarkers of Inflammation in Metabolic Dysfunction–Associated Steatotic Liver Disease

**DOI:** 10.3390/jcm15020559

**Published:** 2026-01-09

**Authors:** Isa Yalcinkaya, Iskender Ekinci, Seyma Dumur, Eda Nur Duran, Hafize Uzun, Melda Yalcinkaya, Elif Kadioglu Yeniyurt, Omer Vehbi Alpaydin, Gulden Anataca, Omur Tabak

**Affiliations:** 1 Department of Hematology, Basaksehir Cam and Sakura City Hospital, Health Sciences University, 34480 Istanbul, Turkey; isayk@hotmail.com; 2 Department of Internal Medicine, BezmialemVakif University, 34093 Istanbul, Turkey; driskenderekinci@gmail.com; 3 Faculty of Medicine, Department of Medical Biochemistry, Istanbul Atlas University, 34403 Istanbul, Turkey; seyma.dumur@atlas.edu.tr (S.D.); huzun59@hotmail.com (H.U.); 4 Department of Internal Medicine, Kanuni Sultan Suleyman Training and Research Hospital, Health Sciences University, 34303 Istanbul, Turkey; enurduran@gmail.com (E.N.D.); ekadioglu93@gmail.com (E.K.Y.); vehbialpaydin@gmail.com (O.V.A.); ganataca@yahoo.com (G.A.); 5 Department of Internal Medicine, Bayrampasa State Hospital, 34040 Istanbul, Turkey; meldagul11@hotmail.com

**Keywords:** activating transcription factor 4, toll-like receptor 4, metabolic dysfunction–associated steatotic liver disease (MASLD), inflammatory pathways

## Abstract

**Background/Objectives:** This study aimed to evaluate the serum activating transcription factor 4 (ATF4) and toll-like receptor 4 (TLR4) levels in patients with metabolic dysfunction–associated steatotic liver disease (MASLD), and to explain the mechanism in the inflammatory and fibrogenic signaling pathways that are thought to play a role in the development of MASLD through these parameters. **Methods:** Eighty-eight patients with MASLD and 88 age-sex matched healthy controls were included in this study. Serum ATF4 and TLR4 concentrations were measured using an ELISA method. **Results:** Both TLR4 (*p* = 0.010) and ATF4 (*p* < 0.001) levels were higher in the MASLD group. In this group, TLR4 showed a negative correlation with age. ROC analysis indicated that an ATF4 value of 1.305 or above identified MASLD with 93.2% sensitivity and 85.2% specificity (AUC = 0.968, *p* < 0.001). For TLR4, a cut-off of 343.5 yielded a sensitivity of 54.5% and a specificity of 70.5% (AUC = 0.613, *p* = 0.01), indicating limited discriminative ability. **Conclusions:** Patients with MASLD had higher serum TLR4 and ATF4 levels, consistent with their involvement in inflammatory and fibrotic pathways. ATF4 showed strong diagnostic performance and may serve as a useful non-invasive marker for early MASLD. When evaluated together with TLR4, it may provide complementary information regarding inflammatory pathway activation.

## 1. Introduction

Non-alcoholic fatty liver disease (NAFLD), recently incorporated into the broader category of metabolic dysfunction–associated steatotic liver disease (MASLD), is characterized by an excessive accumulation of triglycerides within hepatocytes—typically involving more than 5% of the liver parenchyma. The diagnosis still depends on excluding significant alcohol consumption and other well-defined causes of chronic liver injury [1]. Current global estimates suggest that roughly one quarter of the population may be affected, although the distribution is clearly not uniform across regions. For instance, prevalence rates tend to be higher in South America and the Middle East (around 31–32%), while European and African populations show lower figures, approximately 23% and 14%, respectively [2]. MASLD frequently accompanies metabolic disorders such as obesity, insulin resistance, type 2 diabetes, hypertension, and dyslipidemia, underscoring its close relationship with systemic metabolic dysfunction [1,3]. Not surprisingly, the burden is even greater among individuals with T2D. Several analyses, for example, estimate a prevalence of about 55.5% worldwide, reaching nearly 68% in some European cohorts. Moreover, around 37.3% of patients with T2D appear to have non-alcoholic steatohepatitis (NASH), and approximately 17% exhibit advanced fibrosis [4]. These observations together reinforce the notion that MASLD has become an important driver of cardiovascular morbidity, cirrhosis, and hepatocellular carcinoma, and therefore represents a growing public-health challenge [1,3]. Overall, MASLD affects a substantial proportion of the adult population worldwide and represents one of the leading causes of chronic liver disease [5].

Although its pathophysiology has not been fully resolved, MASLD is generally viewed as the outcome of several overlapping processes rather than a single dominant mechanism. Insulin resistance, lipotoxicity, oxidative stress, and inflammation all contribute to disease onset and progression to varying degrees [6,7]. Insulin resistance facilitates the influx of fatty acids into hepatocytes, promoting triglyceride accumulation. As free fatty acids increase, mitochondrial function becomes impaired, eventually leading to oxidative and endoplasmic reticulum (ER) stress. Interestingly enough, these cellular stresses initiate compensatory mechanisms yet simultaneously intensify inflammatory signaling, creating a cycle that amplifies hepatocellular injury [8,9].

Toll-like receptor 4 (TLR4), which recognizes lipopolysaccharides (LPS) and several endogenous ligands, plays a key role in orchestrating innate immune responses. Its activation leads to the production of pro-inflammatory, antiviral, and antibacterial cytokines. In the context of MASLD, increased TLR4 activity is often regarded as an important contributor to hepatic inflammation, possibly through recognition of damage-associated molecular patterns such as heat-shock proteins and fibronectin [10,11]. In contrast, activating transcription factor 4 (ATF4) functions as a central downstream regulator of the PERK arm of the unfolded-protein response. Under conditions of ER oxidative stress, ATF4 becomes upregulated and influences processes related to oxidative stress responses, apoptosis, and fibrogenesis [12].

The simultaneous activation of TLR4-mediated inflammation and ATF4-mediated cellular stress response in MASLD is considered an essential mechanism in the progression of the disease [10,11,12]. Therefore, assessing circulating TLR4 and ATF4 levels may provide a new biochemical approach for the early detection of MASLD and monitoring disease activity. Despite the high global burden of MASLD, there remains a lack of reliable and widely available non-invasive serum biomarkers for early disease detection and risk stratification. Current diagnostic approaches largely rely on imaging techniques or composite clinical scores, underscoring the need for novel circulating biomarkers that reflect underlying inflammatory and cellular stress pathways [13]. In this study, serum TLR4 and ATF4 levels were examined in patients with MASLD to investigate the relationship between these two parameters and the presence and severity of the disease, to elucidate their possible roles in inflammation and fibrogenesis.

## 2. Materials and Methods

### 2.1. Study Design

This single-center prospective case–control study was conducted according to the guidelines of the Declaration of Helsinki. The study protocol was approved by the Health Sciences University Istanbul Kanuni Sultan Suleyman Training and Research Hospital Ethics Committee (number and date of approval: 2021.12.327/8 December 2021). All subjects gave their informed consent for inclusion before they participated in the study.

### 2.2. Subjects

A total of 88 patients with MASLD from Istanbul Kanuni Sultan Suleyman Training and Research Hospital, Department of Internal Medicine, were included in the study. Participants were recruited between August 2022 and November 2022 from the Internal Medicine outpatient clinics of the same hospital. In the control group who were age- and gender-matched with the patients, 88 individuals who were admitted to the check-up center of our hospital for routine controls and those who did not have chronic diseases and active infections were included.

### 2.3. Inclusion Criteria

The patient group consisted of patients with hepatic steatosis (at least grade 2 on abdominal ultrasonography), hepatic steatosis index (HSI) ≥ 36, and non-alcohol-consuming patients. The control group consisted of healthy individuals who had no disease and no hepatic steatosis detected on abdominal ultrasonography.

### 2.4. Exclusion Criteria

Those under 18 years of age, pregnant and breastfeeding women, patients with a history of alcohol consumption, patients with active infection, patients with malignancy, patients on chronic renal failure/hemodialysis/peritoneal dialysis, patients with severe malnutrition, patients with advanced stage (stage 3–4) heart failure, patients with chronic inflammatory diseases, and patients with chronic lung disease (such as chronic obstructive pulmonary disease (COPD), bronchiectasis, asthma, pulmonary hypertension) were excluded from the study. Patients with clinical, laboratory, or ultrasonographic features suggestive of cirrhosis or portal hypertension were excluded from the study.

### 2.5. Data Collection

Age, gender, and smoking status were recorded. Height (m) and weight (kg) of all participants were measured, and body mass index (BMI) was calculated by the formula: weight in kilograms divided by height in meters squared. Patients were classified according to BMI as follows: normal 18.5–24.9 kg/m^2^, overweight 25–29.9 kg/m^2^, class 1 obesity 30–34.9 kg/m^2^, class 2 obesity 35–39.9 kg/m^2^, and class 3 obesity >40 kg/m^2^ [14]. Waist circumference and hip circumference were measured, and the ratio of waist circumference to hip circumference was calculated. Comorbid diseases, including T2D, hypertension, dyslipidemia, and the presence of MetS were noted. MetS diagnosis was made by taking into account the International Diabetes Federation diagnostic criteria: central obesity (waist circumference: ≥94 cm in men, ≥80 cm in women) accompanied by at least two of the following conditions; triglyceride ≥150 mg/dL, high density lipoprotein <40 mg/dL in men/<50 mg/dL in women, blood pressure ≥ 130/85 mmHg, fasting blood glucose ≥ 100 mg/dL or presence of T2D diagnosis [15].

## 3. Parameters

### 3.1. Sample Collection

After fasting for at least 8 h, blood samples were taken from the antecubital vein between 8:00 and 9:00 in the morning from all individuals who agreed to participate in the study and immediately centrifuged at approximately 3000× *g* for 10 min at room temperature. The samples obtained were stored at −80 °C until the study and were dissolved only once for examination. All serum samples were analyzed in the same session, unaware of clinical information. In addition, laboratory personnel performing the ELISA measurements were blinded to the clinical status of the participants to minimize potential measurement bias. All ELISA measurements were performed in duplicate, and mean values were used for statistical analyses. Hemolyzed or lipemic samples were excluded from the analysis to ensure analytical reliability.

### 3.2. Hepatic Steatosis Index (HSI)

HSI is a screening tool that not only indicates the presence of MASLD but also provides information about the degree of the disease. MASLD can be ruled out with an HSI value below 30 (%93.1 sensitivity), whereas an HSI score of 36 or higher strongly suggests its presence (% 92.4% specificity) [16].

Hepatic steatosis index: a simple screening tool reflecting nonalcoholic fatty liver disease. Dig Liver Dis 2010; 42: 503–8.) HSI value is calculated as follows: HSI = 8 × (ALT/AST) + BMI (+2 if T2D, +2 if female).

### 3.3. Assessment of Serum ATF4 Levels

Serum ATF4 levels in patient and control sera were studied with a commercial ELISA kit (Human ATF4 (Activating Transcription Factor 4), E-EL-H0524, Elabscience, Houston, TX, USA). All samples were examined twice. Intra and inter-CV (*n* = 20) for ATF4 were determined to be 8% and 9%, respectively.

### 3.4. Assessment of Serum TLR4 Levels

Serum TLR4 levels in patient and control sera were studied with a commercial ELISA kit (Human ATF4 (Human TLR4 (Toll-Like Receptor 4), E-EL-H6123, Elabscience, USA). All samples were examined twice. Intra and inter-CV (*n* = 20) for TLR4 were determined to be 8% and 9%, respectively.

Biochemical parameters were determined using the enzymatic methods (COBAS 8000, ROCHE-2007, Tokyo, Japan).

Insulin resistance (HOMA-IR) was calculated using the Homeostasis Model Assessment of Insulin Resistance formula: multiply fasting insulin (µU/mL) by fasting glucose (mg/dL), then divide by 405 [17].

### 3.5. Statistical Analysis

SPSS (Statistical Package for the Social Sciences) version 26.0 for Windows (IBM Corporation, Chicago, IL, USA) was used for statistical analysis. Categorical variables were presented as numbers and percentages, and numerical variables were presented as mean ± standard deviation. Chi-square test was used to compare categorical data between groups. The comparison of the two groups regarding numerical variables involved using the Student’s *t*-test for normally distributed data and the Mann–Whitney U test for data that did not follow a normal distribution. Pearson correlation analysis was performed for normally distributed variables, and Spearman correlation analysis was performed for abnormally distributed variables. For the analysis of the association between ATF4 and TLR4 levels with MASLD development, cut-off values were determined by receiver operating characteristic (ROC) analysis. *p* value < 0.05 was considered significant. Normality of continuous variables was assessed using visual (histogram) and analytical methods prior to statistical testing. Group comparisons between patients with MASLD and healthy controls were performed using independent-samples t-tests or Mann–Whitney U tests, as appropriate, depending on data distribution. Categorical variables were compared using the chi-square test. All statistical analyses were conducted to compare groups as defined, without matching for obesity status.

## 4. Results

This study included 88 patients with MASLD and 88 age- and gender-matched healthy individuals. The mean age of all subjects was 40.76 ± 8.34 years. Demographic data, smoking status, anthropometric measurements, blood pressure values, and comorbid diseases of the subjects are presented in Table 1. Weight, body mass index (BMI), waist circumference (WC), hip circumference (HC), and waist-to-hip ratio (WC/HC) were higher in the MASLD patient group than in the control group; additionally, there was a significant difference between the groups when stratified by BMI.

**Figure 1:**
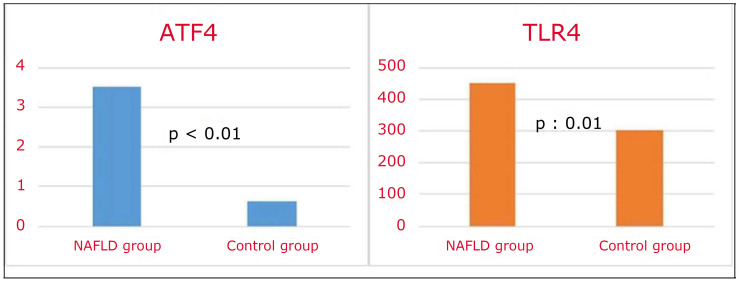
Serum ATF4 and TLR4 levels in MASLD and control groups.

In the MASLD group, glucose, HbA1c, HOMA-IR, AST, ALT, LDL cholesterol, triglyceride, CRP, and platelet count were higher, while HDL cholesterol was lower than in the control group (Table 2). Serum TLR4 and ATF4 levels were found to be higher in the MASLD group than in the control group (Table 2 and Figure 1).

HSI in MAFLD group was calculated as 48.16 ± 6.84 (minimum: 37.89, maximum: 70.45).

In the correlation analysis of serum ATF4 and TLR4 levels with age, weight, BMI, WC, HC, WC/HC ratio and some laboratory parameters (glucose, HOMA-IR, HbA1c, creatinine, ALT, AST, LDL cholesterol, HDL cholesterol, triglyceride, total cholesterol, CRP and platelet count) in the MASLD patient group, it was found that only serum TLR4 levels had a negative significant correlation with age (r: −0.294, *p*: 0.005).

After the patients were grouped according to gender, smoking status, obesity classification, and the presence of comorbid diseases such as DM, HT, HL, and metabolic syndrome, they were compared in terms of serum ATF4 and TLR4 levels, but no difference was observed between the groups. (Table 3).

**Figure 2:**
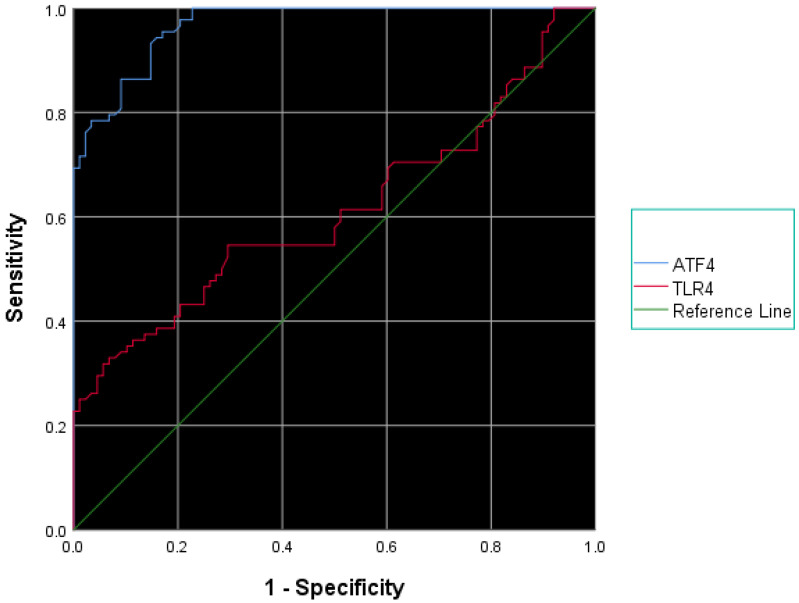
ROC analysis of serum ATF4 and TLR4 levels for the diagnosis of MASLD.

As shown in Figure 2, serum ATF4 level of 1.305 and above [93.2% sensitivity, 85.2% specificity, area under the curve: 0.968 (%95 Cl: 0.947–0.988), *p* < 0.001] and serum TL4 levels of 343.5 and above [54.5% sensitivity, 70.5% specificity, area under the curve: 0.613 (%95 Cl: 0.528–0.697), *p*: 0.01] were determined as cut-off values for the diagnosis of MAFLD (Figure 2).

## 5. Discussion

The most significant findings of this study are the markedly elevated serum levels of ATF4 and TLR4 in patients with MASLD compared to healthy controls, along with their diagnostic utility. The term MASLD is used in accordance with the recently updated, consensus-based nomenclature for steatotic liver disease, developed through an international, multi-stakeholder process involving major liver societies, replacing the former designation of NAFLD. Hereafter, the term MASLD is used for consistency with existing literature. Notably, ATF4 demonstrated strong diagnostic performance, with a serum level of ≥1.305 ng/mL identifying MASLD with 93.2% sensitivity and 85.2% specificity, highlighting its potential as a highly sensitive non-invasive biomarker. While TLR4 levels were also significantly elevated in the MASLD group, their discriminative ability was more modest (cut-off ≥ 343.5 pg/mL). These results underscore the role of inflammatory and stress-response pathways in MASLD development and suggest that serum ATF4 could serve as a reliable early biomarker in clinical practice. The absence of significant associations between these biomarkers and demographic or comorbidity subgroups further supports their potential usefulness regardless of common confounding factors.

MASLD patients in our cohort exhibited typical metabolic risk profiles, including significantly higher rates of obesity, T2D, hypertension (HT), hyperlipidemia (HL), and MetS. Despite the presence of these comorbid conditions, no significant differences in serum TLR4 or ATF4 levels were observed when patients were stratified by sex, smoking status, obesity classification, or comorbid disease presence. This suggests that both TLR4 and ATF4 elevations are closely related to MASLD itself and not confounded by other demographic or metabolic factors. Anthropometric parameters, including BMI, WC, HC, and WC/HC, were significantly elevated in the MASLD group, consistent with previous studies demonstrating a strong association between obesity and MASLD [18,19]. Obesity is a major component of the metabolic syndrome and has been shown to contribute directly to hepatic steatosis through mechanisms involving increased free fatty acid (FFA) flux, insulin resistance, and proinflammatory cytokine production in MASLD [20].

Our study also revealed significant elevations in glycemic markers fasting glucose, glycated hemoglobin (HbA1c), and HOMA-IR in the MASLD group compared to controls, in line with literature suggesting a strong relationship between insulin resistance and MASLD pathogenesis [4,21]. Insulin resistance contributes to steatotic lipid accumulation by enhancing lipogenesis, impairing β-oxidation, and promoting inflammatory responses within the liver. Dyslipidemia is another hallmark of MASLD. Our findings of elevated serum LDL cholesterol, triglycerides, and total cholesterol, along with reduced HDL cholesterol levels in MASLD patients, are consistent with earlier reports [22,23]. These lipid abnormalities exacerbate hepatic steatosis and may accelerate progression to non-alcoholic steatohepatitis (NASH), fibrosis, and cirrhosis.

Low-grade chronic inflammation has emerged as a key mechanistic component driving hepatocellular damage and disease progression in MASLD [24]. Pro-inflammatory cytokines such as TNF-α, IL-1β, and IL-6 are elevated in MASLD and contribute to hepatocellular injury, fibrosis, and progression to NASH [25]. C-reactive protein (CRP), a widely used acute-phase reactant, is a reliable systemic marker of low-grade inflammation. Consistent with previous studies [26,27,28], our results showed significantly higher CRP levels in MASLD patients compared to controls, reinforcing the inflammatory basis of MASLD. The role of platelet count in MASLD is still debated. While some studies have reported no association [29], others have found platelet counts to be either elevated [30] or decreased [31] depending on disease stage. In our study, platelet counts were significantly higher in MASLD patients. Given that liver biopsy was not performed, and non-invasive fibrosis markers were not applied, this increase may reflect an early inflammatory state rather than advanced fibrosis. Platelet levels may decrease in later stages of liver damage due to reduced thrombopoietin production or splenic sequestration; thus, further research with histological confirmation is warranted.

### 5.1. Role of TLR4 in Inflammation

TLR4 is a type I transmembrane protein that plays a critical role in innate immunity by recognizing LPS and FFAs both elevated in obesity and MASLD. Its activation promotes NF-κB-mediated transcription of proinflammatory genes [32]. Experimental studies have shown that increased TLR4 signaling contributes to liver inflammation and fibrogenesis in MASLD and NASH models [33,34,35]. In our study, TLR4 levels were significantly elevated in the MASLD group, and a negative correlation with age was observed, which is consistent with prior findings suggesting decreased TLR4 expression or responsiveness with aging [36,37].

Recent studies have highlighted the central role of TLRs, particularly TLR4, in the development of obesity-related metabolic disturbances. TLR4 activation, in response to elevated circulating free fatty acids (FFAs) and endotoxins, promotes chronic low-grade inflammation, insulin resistance, and hepatic lipid accumulation, all of which are key features of MASLD. Campos-Bayardo et al. emphasized that TLR-mediated signaling pathways are significantly up-regulated in obese individuals and contribute to the progression of MetS and its hepatic manifestations, including MASLD [10].

Moreover, elevated TLR4 expression has been associated with insulin resistance, β-cell dysfunction, and progression of type 2 diabetes [38,39,40]. Our results support the hypothesis that TLR4 contributes to the pathophysiological mechanisms underlying both MASLD and metabolic dysfunction. However, TLR4 levels did not differ by the presence of diabetes, MetS, or obesity class in our study, indicating a more universal role across various metabolic phenotypes. Although TLR4 demonstrated limited discriminative ability (AUC = 0.613), its elevation in MASLD underscores the importance of innate immunity and inflammation in disease pathogenesis. TLR4 activation by lipopolysaccharides and free fatty acids is known to trigger NF-κB–mediated cytokine release, promoting hepatic inflammation and fibrosis. The observed negative correlation between age and TLR4 may suggest age-related modulation of innate immune response or a higher inflammatory drive in younger MASLD patients.

### 5.2. Diagnostic and Pathophysiological Significance of ATF4

ATF4 is a key transcription factor activated primarily through the PERK branch of the unfolded protein response (UPR) during endoplasmic reticulum (ER) stress. It regulates a broad range of cellular processes, including oxidative stress responses, inflammation, apoptosis, and lipid metabolism. In the liver, sustained activation of ATF4 under metabolic stress promotes lipogenesis, oxidative injury, and hepatocyte death, thereby contributing to the progression of MASLD. ATF4 is also a central regulator of the ER stress response and the unfolded protein response (UPR), both of which are closely linked to MASLD pathogenesis. In hepatocytes, ATF4 promotes lipogenesis, oxidative stress, and apoptosis under chronic metabolic overload [41]. In this study, serum ATF4 levels were significantly elevated in MASLD patients, reflecting systemic ER stress and early hepatic injury. The diagnostic performance of ATF4 was remarkably high (AUC = 0.968, sensitivity = 93.2%, specificity = 85.2%), underscoring its potential as a reliable biomarker for MASLD detection. Consistent with previous reports in diet-induced obese mice, increased hepatic ATF4 expression has been associated with upregulation of inflammatory mediators such as LECT2 and enhanced activation of downstream effectors including CHOP, SREBP-1c, and autophagy-related pathways [42].

Interestingly, like TLR4, ATF4 concentrations did not differ significantly among clinical subgroups, suggesting that ATF4 may represent a universal indicator of metabolic stress–induced hepatic dysfunction. Overall, these findings highlight ATF4 as both a mechanistic mediator linking ER stress to hepatic inflammation and a promising diagnostic marker in MASLD.

Importantly, receiver operating characteristic (ROC) analysis showed that a serum ATF4 level of ≥1.305 ng/mL predicted MASLD with 93.2% sensitivity and 85.2% specificity, indicating excellent diagnostic performance. By contrast, TLR4 levels ≥ 343.5 pg/mL provided only moderate diagnostic value (sensitivity: 54.5%, specificity: 70.5%. These findings suggest that ATF4 is a more reliable biomarker for MASLD detection and could potentially serve as a non-invasive alternative to current imaging-based methods.

### 5.3. Study Impact and Future Directions

These findings highlight the potential clinical utility of serum ATF4 as a novel biomarker for early MASLD detection and disease monitoring. Future longitudinal studies with larger cohorts are warranted to confirm these results, explore the mechanistic link between ATF4-TLR4 signaling and hepatic fibrosis, and evaluate the response of these markers to therapeutic interventions (e.g., weight loss, insulin sensitizers, or anti-inflammatory agents). In summary, this study identifies ATF4 as a robust and sensitive marker reflecting ER stress–induced hepatocellular dysfunction, while TLR4 indicates the parallel activation of inflammatory pathways. Their combined evaluation may further contribute to the understanding of MASLD-related inflammatory pathways.

### 5.4. Limitations

This study has several limitations that should be acknowledged. The diagnosis of MASLD relied on ultrasonography and the hepatic steatosis index (HSI), without histological confirmation by liver biopsy; therefore, liver fibrosis stage could not be evaluated, and the presence of steatohepatitis could not be definitively established. In addition, elastography-based assessments, such as shear wave elastography (SWE) and the controlled attenuation parameter (CAP), were not performed, which may have limited the assessment of liver stiffness and steatosis severity. Non-invasive fibrosis scoring systems (e.g., FIB-4, APRI, MASLD fibrosis score) were not applied, as the primary objective of the study was to assess the diagnostic performance of ATF4 and TLR4 for MASLD detection rather than fibrosis stratification; however, this limits conclusions regarding associations with disease severity. Furthermore, the potential effects of anti-inflammatory, antidiabetic, or lipid-lowering medications on circulating ATF4 and TLR4 levels were not specifically evaluated. Finally, the cross-sectional design precludes causal inference or evaluation of temporal changes. Future studies incorporating histopathological validation, comprehensive fibrosis assessment, and longitudinal follow-up in larger, well-characterized cohorts are needed to confirm and extend these findings.

In conclusion, this study demonstrates that serum ATF4 levels are markedly increased in individuals with MASLD, while TLR4 elevation appears to reflect activation of inflammatory pathways. Among the two biomarkers, ATF4 exhibited superior diagnostic accuracy, characterized by high sensitivity and specificity, highlighting its potential utility as a non-invasive screening biomarker for early MASLD detection. Given the limitations of current diagnostic approaches, including imaging modalities and the invasiveness of liver biopsy, ATF4 may represent a valuable adjunct for identifying at-risk populations, particularly individuals with obesity, insulin resistance, or metabolic syndrome. Although TLR4 remains biologically relevant to MASLD development, its moderate diagnostic performance limits its role as a standalone diagnostic marker. Future large-scale, longitudinal studies incorporating histopathological validation, fibrosis staging, and extended clinical follow-up are needed to confirm these findings, assess their prognostic significance, and evaluate the potential roles of ATF4 and TLR4 in disease monitoring, therapeutic response assessment, and personalized management strategies across the MASLD spectrum.

## Figures and Tables

**Table 1:** Demographic data of the subjects.

		MASLD Group (*n* = 88)	Control Group (*n* = 88)	*p*
Age, year		41.09 ± 6.9	40.44 ± 9.6	0.608
Male, *n* (%)		31 (35.2)	30 (34.1)	0.874
Smoker, *n* (%)		39 (44)	27 (30)	0.062
Weight, kg		94.24 ± 18.92	67.40 + 11.62	<0.001
BMI, kg/m^2^		35.42 ± 6.6	23.87 ± 3.1	<0.001
	normal, *n* (%)	-	57 (64.7)	<0.001
	overweight, *n* (%)	15 (17)	31 (35.2)	
	class-1 obesity, *n* (%)	41 (46.5)	-	
	class-2 obesity, *n* (%)	16 (18.1)	-	
	class-3 obesity, *n* (%)	16 (18.1)	-	
WC, cm		113.81 ± 12.06	82.77 ± 10.98	<0.001
HC, cm		119.02 ± 12.99	98.03 ± 8.52	<0.001
WC/HC ratio		0.96 ± 0.077	0.84 ± 0.076	<0.001
SBP, mmHg		126.81 ± 8.61	-	**-**
DBP, mmHg		75 ± 5.12	-	**-**
DM, *n* (%)		32 (36.3)	-	**-**
HT, *n* (%)		20 (22.7)	-	**-**
HL, *n* (%)		50 (56.8)	-	**-**
Metabolic Syndrome, *n* (%)		51 (57.9)	-	**-**

Abbreviations: BMI: Body mass index, WC: waist circumference, HC: hip circumference, DM: Type-2 diabetes mellitus, HT: Hypertension, HL: Hyperlipidemia.

**Table 2:** Analysis of laboratory parameters between groups.

Parameter	MASLD Group (*n* = 88)	Control Group (*n* = 88)	*p*
Glucose (mg/dL)	116.9 ± 47.34	90.16 ± 7.54	<0.001
HOMA-IR	6.85 ± 6.41	2.24 ± 1.09	<0.001
HbA1c (%)	6.45 ± 1.49	5.21 ± 0.29	<0.001
Creatinine (mg/dL)	0.72 ± 0.17	0.71 ± 0.16	0.736
AST (U/L)	27.05 ± 16.87	16.87 ± 5.16	<0.001
ALT (U/L)	36.66 ± 26.89	16.03 ± 8.99	<0.001
LDL (mg/dL)	116.93 ± 35.66	102.97 ± 29.01	0.014
HDL (mg/dL)	45.01 ± 9.57	55.74 ± 11.25	<0.001
Triglyceride (mg/dL)	180.78 ± 96.91	100.64 ± 55.40	<0.001
Total Cholesterol (mg/dL)	198.16 ± 39.68	178.83 ± 31.66	0.002
CRP (mg/L)	5.63 ± 6.39	1.65 ± 1.48	<0.001
Platelet count (10^3^/µL)	295.19 ± 98.13	257.64 ± 58.47	0.001
ATF4 (ng/mL)	3.53 ± 2.65 (2.7)	0.64 ± 0.47 (0.43)	<0.001
TLR4 (pg/mL)	454.7 ± 298.6	303.3 ± 157.8	0.010

HOMA-IR: Homeostatic Model Assessment, HbA1c: Glycosylated hemoglobin, AST: Aspartate transaminase, ALT: Alanine transaminase, LDL: Low-density lipoprotein, HDL: High-density lipoprotein, CRP: C-reactive protein, ATF4: Activating transcription factor 4, TLR4: Toll-like receptor 4.

**Table 3:** Analysis of serum ATF4 and TLR4 levels in subgroups.

		ATF4	*p*	TLR4	*p*
Gender	Male, *n* = 31	3.27 ± 2.53	0.402	432.5 ± 291.3	0.437
	Female, *n* = 57	3.67 ± 2.72		466.7 ± 304.4	
Smoker	Yes, *n* = 39	3.47 ± 2.62	0.860	426.2 ± 291.2	0.465
	No, *n* = 49	3.57 ± 2.69		477.4 ± 305.5	
Type 2 diabetes mellitus	Yes, *n* = 32	3.59 ± 2.57	0.706	506.1 ± 329.2	0.218
	No, *n* = 56	3.49 ± 2.71		425.3 ± 278.5	
Hypertension	Yes, *n* = 20	3.13 ± 2.18	0.921	412.1 ± 286.6	0.537
	No, *n* = 68	3.64 ± 2.77		467.2 ± 303	
Hyperlipidemia	Yes, *n* = 50	3.04 ± 1.95	0.490	422.8 ± 296	0.354
	No, *n* = 38	4.17 ± 3.27		496.6 ± 300.8	
Metabolic Syndrome	Yes, *n* = 51	3.45 ± 2.54	0.879	449.4 ± 298.5	0.949
	No, *n* = 37	3.63 ± 2.82		461.9 ± 302.8	
Obesity Classification	Overweight, *n* = 15	4.61 ± 3.45	0.722	528.8 ± 300.9	0.540
	Class-1, *n* = 41	3.12 ± 2.20		428.6 ± 301.2	
	Class-2, *n* = 16	3.75 ± 3.13		403.5 ± 277.9	
	Class-3, *n* = 16	3.32 ± 2.24		503.1 ± 317.7	

## Data Availability

The datasets used and analyzed in this study are available from the corresponding authors upon reasonable request.
